# Anti-Vascular endothelial growth factor therapy impairs endothelial function of retinal microcirculation in colon cancer patients – an observational study

**DOI:** 10.1186/2040-7378-5-7

**Published:** 2013-05-13

**Authors:** Manja Reimann, Gunnar Folprecht, Rocco Haase, Karolin Trautmann, Gerhard Ehninger, Heinz Reichmann, Focke Ziemssen, Tjalf Ziemssen

**Affiliations:** 1 Autonomic and neuroendocrinological laboratory, Center of Clinical Neuroscience, University Hospital Carl Gustav Carus, Dresden, Fetscherstr 74, Germany; 2 Department of Neurology, University Hospital Carl Gustav Carus, Dresden, Germany; 3 Department of Internal Medicine I, University Cancer Center, University Hospital Carl Gustav Carus, Dresden, Germany; 4 Center for Ophthalmology, Eberhard Karl University Tuebingen, Tuebingen, Germany

**Keywords:** Angiogenesis, Endothelium, Microcirculation, Cerebral hemodynamics, Nitric oxide

## Abstract

**Background:**

To assess acute effects of bevacizumab (anti-VEGF therapy) on cerebral microvessels and systemic cardiovascular regulation.

**Design and subjects:**

20 consecutive patients with colorectal cancer (median age: 60.4 years, range 45.5-73.9 years) received bevacizumab intravenously (5 mg/kg) uncoupled of chemotherapy. Prior to and within the first 24 hours after bevacizumab infusion, patients were investigated for retinal endothelial function. A series of a triple 24-hour ambulatory blood pressure measurement was conducted. Retinal endothelial function was determined as flicker light-induced vasodilation. The integrity of baroreflex arc and autonomic cardiovascular control was examined by stimulatory manoeuvres.

**Results:**

Bevacizumab therapy significantly reduced the vasodilatory capacity of retinal arterioles in response to flicker light. A slight decrease in diastolic pressure and heart rate was observed after bevacizumab infusion but this was unrelated to changes in retinal function. The pressure response upon nitroglycerin was largely preserved after bevacizumab infusion. The proportion of patients with abnormal nocturnal blood pressure regulation increased under anti-angiogenic therapy. Autonomic blood pressure control was not affected by bevacizumab treatment.

**Conclusions:**

Bevacizumab acutely impairs microvascular function independent of blood pressure changes. Imaging of the retinal microcirculation seems a valuable tool for monitoring pharmacodynamic effects of bevacizumab.

**Trial registration:**

NCT ID: NCT00740168

## Background

Vascular endothelial growth factor (VEGF) is a key mediator of physiological and pathological angiogenesis. As all solid tumors are dependent on pathological angiogenesis, anti-VEGF therapy has been approved in various advanced and metastatic cancer types [1,2].

The rationale for treating cancer patients with angiogenesis inhibitors has been on the assumption that unlike in tumor vessels survival and functioning of the normal vasculature is largely independent of VEGF signalling [3]. Although clinical trials have proven the clinical benefit of bevacizumab and other angiogenesis inhibitors for the treatment of advanced cancers [1,2,4,5], their use has been associated with hypertension and thromboembolic events [5-8]. Several mechanisms of the underlying cardiovascular toxicity have been proposed including the inhibition of vasomotor effects by VEGF but also structural and functional microvascular rarefaction [9-11]. Accordingly, changes in endothelial function have been observed in cancer patients treated by angiogenesis inhibitors [10,11] and may serve as an important clinical marker for cardiovascular complications and therapy outcome. This hypothesis is corroborated by multiple clinical trials showing an association between bevacizumab-induced hypertension and response to therapy [12].

Here, we investigated the acute impact of bevacizumab administration on retinal vessel reactivity in relation to acute changes of cardiovascular regulation inclusive blood pressure using the dynamic retinal vessel analysis and evaluation of the autonomic nervous system. We hypothesised that the dynamic retinal vessel analysis represents a useful non-invasive and easy applicable tool to monitor functional microvascular alterations following VEGF inhibition.

## Materials and methods

### Participants and ethics

Patients with histological confirmed metastatic or locally advanced, inoperable colorectal cancer were enrolled into the study. All patients had an indication for treatment with bevacizumab / fluoropyrimidine containing chemotherapy regimens and had not previously received drugs directly targeting the VEGF pathway. Bevacizumab was administered at a dose of 5 mg/kg one day before the start of chemotherapy (day 0). To separate effects, fluoropyrimidine based chemotherapy was started after completion of all functional vascular measurements on day 1 as described below. Patients were evaluated by dynamic retinal vessel analysis, autonomic function tests and 24-hour ambulatory blood pressure monitoring (ABPM) the day before (day -1) and the day after bevacizumab infusion (day 1). ABPM was also applied on the day of bevacizumab treatment (day 0). The protocol was approved by the Ethics Review Board of the Medical Faculty of the Dresden University of Technology. All patients signed a written informed consent before inclusion into the study. The procedures conformed to the principles of the Declaration of Helsinki (Clinical Trials Registration NCT ID: NCT00740168)

### Evaluation of the autonomic nervous system

All measurements were performed in a temperature and humidity controlled specialized autonomic laboratory. Consumption of caffeine or other xanthine containing foods were not permitted on the examination day. Alcohol and tobacco usage was ceased at the day preceding the measurements. For continuous cardiovascular monitoring using the SUEMPATHY device (Suess Medizin-Technik, Aue, Germany) including the non-invasive blood pressure monitoring CBM3000 device (Nihon Colin Co, Komaki, Japan) the patient was placed in the supine position on a tilt table [13]. After a resting period of 20 minutes the following manoeuvres were performed. First blood pressure and heart rate responses to sublingual administration of 400 μg glyceroltrinitrate (Nitrolingual^®^ akut Spray, Pohl Boskamp, Hohenlockstedt, Germany) were recorded for five minutes followed by deep metronomic breathing at a rate of six breaths per minute for two minutes. For evaluation of orthostatic reaction, patients were tilted to a 60° upright position for 15 minutes. After 10 minutes of head-up tilting, 400 μg glyceroltrinitrate was repeatedly administered while tilting was continued for another five minutes. During head-up tilting the subsequent decrease in ventricular filling and stroke volume evokes a transient fall in arterial pressure which is counter-regulated by the baroreflex arc with resultant tachycardia (vagal withdrawal) and vasoconstriction (sympathetic activation). This reaction is amplified by the application of vasodilatory agents such as glyceroltrinitrate. Orthostatic hypotension may occur as a result of sympathetic dysfunction which is defined as a reduction in systolic blood pressure of at least 20 mmHg and/or diastolic pressure of at least 10 mmHg within three minutes of upright tilt [14].

### Retinal vessel analysis

The Dynamic Retinal Vessel Analyzer (DVA, IMEDOS GmbH, Jena, Germany) was used for digital fundus imaging to perform static and dynamic retinal vessel analysis. Thirty minutes after initiation of mydriasis of the right pupil using 1% tropicamide eye drops, each participant underwent seven-field fundus photography using the system Visualis (IMEDOS, FF450plus, 535–561 nm, 30° image, 1840×1360 pixel) as previously described [15]. The arteriolar-to-venular ratio (AVR) was calculated from the central retinal arterial and venous equivalents to quantify generalized arterial narrowing. Dynamic vessel analysis was performed allowing non-invasive diagnosis of microvascular function by measuring the diameter of retinal arteries and veins continuously [16,17]. An optoelectronic shutter was inserted in the retinal camera which interrupted the observation light with a frequency of 12.5 Hz. The baseline diameter was measured for 30 seconds in continuous light followed by three cycles of 20-second flicker provocation and 50-second steady fundus illumination. An arterial and venous segment of approximately 1.5 mm length was subsequently evaluated. An interval of 30 seconds before each flicker stimulation was considered as baseline to which the subsequent diameter response was normalised. The peak dilation was the largest vessel diameter at the end of each flicker stimulation averaged across three flicker periods. Changes in ocular hemodynamic parameters were expressed as percent change over baseline values.

### 24-h Ambulatory blood pressure monitoring

Validated automated 24-hour blood pressure devices (Boso GmbH, Jungingen, Germany) fulfilling the accuracy criteria of the Association for the Advancement of Medical Instrumentation were used to measure blood pressure and heart rate at intervals of 15 minutes at daytime (6–22 hours) and of 30 minutes at night (22–6 hours) at three different occasions. For each 24-hour measurement, blood pressures were evaluated separately for daytime and nighttime phases. Arterial daytime hypertension was diagnosed according to the criteria of the European Society of Hypertension (systolic blood pressure ≥ 140 mmHg and/or diastolic blood pressure ≥ 90 mmHg). Nocturnal hypertension was defined according to the guidelines of the American Hypertension Association (systolic blood pressure > 120 mmHg and diastolic blood pressure > 75 mmHg). A non-dipping blood pressure profile was existent when the nocturnal blood pressure fall was less than 10% of daytime values [18,19].

### Multiple trigonometric regressive spectral analysis

Frequency analysis allows for quantification of cardiovascular regulation by assessing spontaneous oscillations in systemic arterial pressure and heart rate. Multiple trigonometric regressive spectral analysis (MTRS, ANS Consult, Freital, Germany) was applied to corresponding values of systolic blood pressure and of R-R interval derived from electrocardiogram. A non-artifactual global data segment of 2 min was analyzed. The local time window was set at 30 seconds and was shifted beat by beat for temporal determination of frequency and amplitude [20,21]. Two main spectral bands are usually considered: High frequency (HF) oscillations (spectral band between 0.15 and 0.4 Hz) of heart rate relate to respiratory sinus arrhythmia and, therefore, to parasympathetic cardiovagal tone. The other oscillation of interest is in the low-frequency range (spectral band between 0.04 and 0.15 Hz), usually centred around 0.1 Hz (six cycles min^-1^). Low-frequency oscillations of heart rate (LF R-R) are thought to reflect the baroreflex-mediated adjustments to the sinus node while LF variations of systolic blood pressure are primarily the result of sympathetically mediated fluctuations in peripheral vasomotor tone. The LF/HF ratio allows for quantification of the relation between the two branches of the autonomic nervous system. The total spectral power describes the amount of variability of a signal or stochastic process at a specific frequency. Baroreflex sensitivity was calculated as the slope of the linear regression line of coherent pairs of the detected oscillations of R-R interval and systolic blood pressure (cross correlation coefficient > 0.7) [20,22,23]. The estimation of a global BRS index was based on the weighted mean of all 300–500 individual BRS values derived from all local data segments analysed according to a variance ratio: (variance reduction of RRI/ variance reduction of SBP)^2^.

### Statistical methods

The SPSS package version 17.0 (SPSS Inc., Chicago, IL, USA) was used for all statistical computations. Data are expressed as mean and standard deviation or quartiles. Assuming non-Gaussian distribution, the acute effects of bevacizumab treatment on hemodynamic parameters were analyzed using Wilcoxon and Friedman Test. Spearman correlation coefficients were calculated between changes of continuous variables. Nominal data were compared by Fisher’s exact test using cross-tabulation. The overall survival of patients was calculated by the methods of Kaplan-Meier. Patients alive were censored at the day of last contact. A two-tailed *p*-value of < 0.05 was used to indicate significance.

## Results

Twenty patients (15 men and 5 women) were enrolled into the study. Eleven patients had rectal and nine colon cancer. The median age was 60.4 [range 45.5-73.9] years. Overall survival of all patients was 23.5 [15.0 – 31.8] months (median [95% CI]). Patients receiving bevacizumab as first line treatment (n = 9) had an average survival of 27.1 [21.8 – 32.4] months and those receiving the antibody as second line treatment (n = 11) survived on average 21.2 [14.0 – 28.5] months.

### Retinal vessel analysis

**Figure 1:**
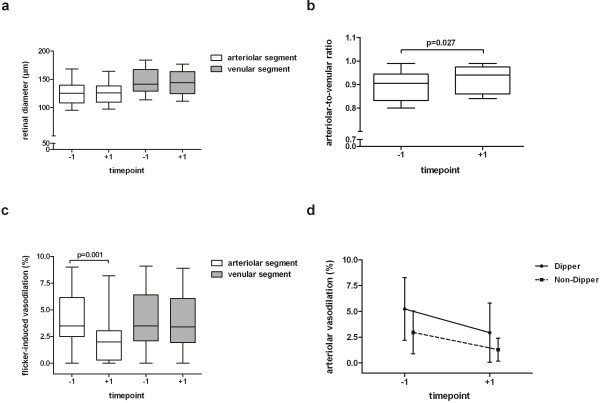
**a-d Functional and structural changes of retinal vessels in colorectal cancer patients in response to bevacizumab infusion. **While the diameter of retinal vessels were unaffected by bevacizumab treatment (Figure 1**a**) it evoked an increase in arteriolar-to-venular ratio (Figure 1**b**) and a decrease in flicker-induced arterial vasodilation (Figure 1**c**). The reactivity of retinal arteries to flickering light tended to be lower in patients with a pathological nocturnal blood pressure profile (non-dippers) than in those with physiological nocturnal blood pressure profile (dippers) (Figure 1**d**). *Legend: *before (day-1) and after (day + 1) bevacizumab infusion.

Static retinal vessel analysis revealed that there was no arteriolar narrowing based on age-normalised reference values of the ARIC (Atherosclerosis Risk in Communities) population. With bevacizumab, no change was observed for arterial diameter, but there was a trend towards a smaller venous diameter (day-1: 146 ± 22 μm vs. day+1: 143 ± 22 μm, p = 0.062, Figure 1a) resulting in a significantly increased AVR after bevacizumab administration (day-1: 0.89 ± 0.07 vs. day+1: 0.92 ± 0.06, p = 0.027, Figure 1b). The arterial response to flicker provocation deteriorated from 4.2 ± 2.7% to 2.2 ± 2.2% within 24 hours of VEGF inhibition while maximal venous dilation remained unaffected (Figure 1c).

### 24 h ABPM profile

**Figure 2:**
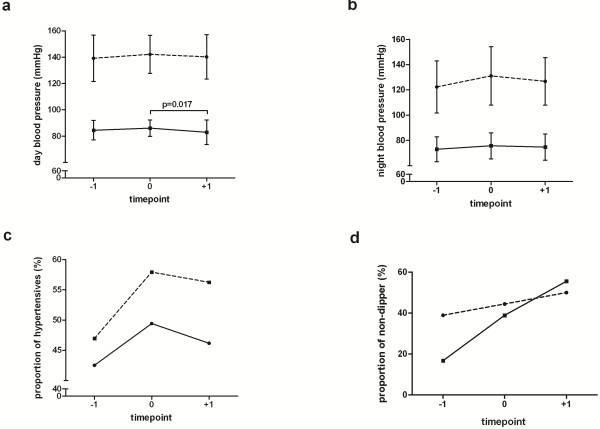
**a-d 24-h ambulatory blood pressure profiles in colorectal cancer patients in response to bevacizumab infusion. **Diastolic blood pressure during daytime but not during nighttime decreased after bevacizumab infusion (Figure 2**a**,**b**). The proportion of individuals with pathological blood pressure profile increased after bevacizumab (Figure 2**c**,**d**). *Legend:* systolic blood pressure (broken lines), diastolic blood pressure (smooth lines); proportions are based on the diagnostic criteria of the European Society of Hypertension and the American Hypertension Association before (day-1), during (day 0) and after (day+1) bevacizumab infusion.

The profiles of 24-h ABPM over three recording days are depicted in Figures 2a and 2b. There was a significant reduction of diastolic pressure after bevacizumab administration (Figure 2a). Twenty-four hour heart rate steadily decreased over time (day -1: 80 ± 12 bpm; day 0: 76 ± 11 bpm; day +1: 74 ± 12 bpm; Friedman p = 0.047). All other parameters were not affected by bevacizumab. There was a trend towards a higher proportion of patients with a hypertensive blood pressure profile under VEGF inhibition (Figure 2c). The proportion of patients with non-dipping blood pressure at night increased continuously over the three recording days (Figure 2d). Although not statistically significant, there was a trend towards a lower retinal arterial reactivity in non-dippers versus dippers irrespective of bevacizumab infusion (p > 0.05, Figure 1d). Decrements of diastolic blood pressure from day 0 to day +1 were neither associated with changes in retinal vascular function (r = -0.250, p = 0.332) nor with AVR (r = -0.422, p = 0.298).

### Autonomic testing

**Figure 3:**
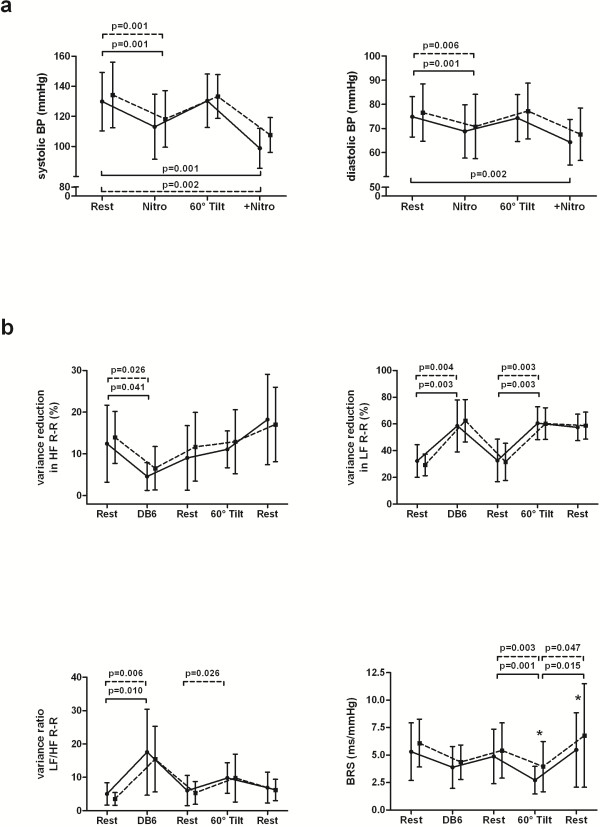
**a-b Blood pressures and frequency bands of R-R interval during autonomic testing in response to bevacizumab infusion. **The blood pressure response to the vasodilator nitroglycerin was largely preserved except for diastolic blood pressure during passive standing (Figure 3**a**). Cardiovascular perturbation by deep breathing and head-up tilt evoked a physiological shift from high to low frequency oscillations in R-R interval which was unaffected by bevacizumab treatment (Figure 3**b**). Adequate adjustments of baroreflex sensitivity were only observed in response to postural change but not to deep breathing irrespective of bevacizumab treatment. *Legend: *BP, blood pressure; BRS, baroreflex sensitivity; DB6, deep breathing at six cycles per minute; HF, high frequency oscillations; LF, low frequency oscillations; Nitro, nitroglycerin; +Nitro, Nitroglycerin application during head-up tilt before (smooth lines) and after (Broken lines) bevacizumab infusion.

Sublingual nitroglycerin (Nitro) evoked similar decreases in systolic blood pressure before and after bevacizumab infusion during supine (rest vs. nitro: p = 0.001 at day-1, p = 0.001 at day+1) and upright position (rest vs. +nitro: p = 0.001 at day-1, p = 0.002 at day+1) (Figure 3a). The nitroglycerin-mediated fall in diastolic blood pressure during head-up tilt (+nitro) was diminished after bevacizumab treatment (rest vs. +nitro, p = 0.002 at day-1; p > 0.05 at day+1). Controlled breathing at six cycles per min resulted in shifts from high to low frequency oscillations of R-R interval (LF/HF ratio of R-R interval: p = 0.010 at day -1; p = 0.006 at day+1) which was not accompanied by adequate adjustments in cardiovagal baroreflex sensitivity (Figure 3b) as observed in healthy individuals [23]. Orthostatic challenge induced significant increases in low frequency oscillations of R-R interval (p = 0.003 at day-1; p = 0.004 at day+1) which were paralleled by an expected decrease in baroreflex sensitivity (p = 0.001 at day-1; p = 0.003 at day+1). Cardiovagal outflow as indexed by high frequency oscillations of R-R interval remained unchanged during orthostasis. Bevacizumab treatment did not modify autonomic adjustments of blood pressure control during cardiovascular perturbation.

## Discussion

Many efforts have been made to target the VEGF pathway in tackling cancer growth and tumor metastasis. Anti-angiogenic therapies using anti-VEGF antibodies or inhibitors of VEGF receptor 2 are increasingly employed in combination with standard therapy for the treatment of advanced cancer. However, predictive markers of efficacy as well as of toxicity have not yet been established. Imaging studies using diffusion contrast enhanced MRI (DCE-MRI) have documented very early changes in response to treatment with VEGF inhibitors [24,25]. However, DCE-MRI technique currently seems too complex and expensive to be incorporated into large multicenter trials [26].

Arterial hypertension is a candidate prognostic biomarker of response to VEGF therapy [27]. One hypothesis is that VEGF inhibitors induce arterial hypertension by increasing vasoconstriction through alteration of nitric oxide (NO) bioavailability. Our method of dynamic analysis of flicker-light induced vasodilation, originally established for application in ophthalmological and cardiovascular research, was therefore used to describe the acute impact of bevacizumab administration on non-invasively measured retinal endothelial function in relation to blood pressure. We clearly demonstrate that anti-angiogenic treatment of colorectal cancer acutely reduces flicker-light induced vasodilation of retinal arteries independent of blood pressure changes. Furthermore, blood pressure responses to a nitrovasodilator were not altered by bevacizumab.

The vasodilatory response of retinal vessels to diffuse luminance flicker stimulation is considered to be endothelium dependent through release of NO [28]. Hence, a diminished vasodilatory capacity in response to flicker-light stimulation may indicate endothelial dysfunction. In line with our results, endothelial dysfunction was also reported by two other trials in cancer patients receiving either bevacizumab or telatinib - a small molecule angiogenesis inhibitor [10,11]. In these trials endothelial function was determined by laser Doppler fluxmetry using pilocarpine [10] and flow-mediated vasodilation of the brachial artery [11]. Although these methods measure endothelial function in different vascular beds it seems that the detrimental effect of angiogenesis inhibitors on endothelial function is not confined to a certain vascular bed and that arterial endothelial dysfunction is a general phenomenon of pharmacological angiogenesis inhibition. This is not surprising considering that VEGF receptor 2, which confers the hypotensive actions of VEGF [29], is virtually expressed by all endothelial cells.

It is however intriguing that we did not observe any functional change in retinal veins after bevacizumab administration. The lack of effect may be due to a marked regional and segmental heterogeneity in vascular endothelial function [30]. Accordingly, it has been reported that shear stress elicits a smaller NO-dependent vasodilation in veins and venules than in their arterial counterparts even on the same segment [31]. Therefore, the extent of possible changes in endothelium-dependent vasodilation induced by any stimulus may be inherently less pronounced in the venous system. Furthermore, it is not fully clear whether the endothelial NO-synthase is also modulated by VEGF signaling in the venous circulation and whether VEGF binding to its respective receptors activates the same molecular machinery than in the arterial system. Although VEGF receptors have been identified on endothelial cells of veins a differential pattern of signal transduction has been suggested along the vascular tree [32].

Although elevated blood pressure or hypertension has been the most frequently documented side effect of anti-angiogenic treatment [33] we did not observe meaningful changes in blood pressure nor in short-term blood pressure regulation by anti-angiogenic therapy. The most probable explanation for this observation is that the frequency of blood pressure elevation is a function of treatment duration [34]. From a range of phase II clinical trials the median interval to incident hypertension was estimated at 4.5 - 6 months from initiation of bevacizumab therapy [35,36]. Under the assumption that there is a progressive evolution of hypertension during chronic VEGF inhibition our findings may imply that endothelial dysfunction is the first step in a sequence of deleterious events evoked by VEGF blockade subsequently evolving into clinical disease [10,11]. *In vitro* and *in vivo* studies clearly demonstrated the role of VEGF in maintaining baseline vascular tone by regulating NO synthesis and prostacyclin release [37]. Loss of action by blockade of VEGF signaling renders endothelial dysfunction highly possible. Consistent with our above postulation, we observed a higher frequency of an abnormal blood pressure profile at night after bevacizumab administration. Furthermore, non-dipping status after anti-angiogenic therapy tended to be related to a stronger impairment of microvascular function. Although the latter data were not statistically significant it still may support our hypothesis of a central role of endothelial dysfunction in the development of VEGF-induced hypertension.

We must acknowledge limitations of our pilot study. The small number of patients precluded us from performing multivariate modeling for analysis of possible interactions, especially co-morbidities, nor from doing correlation analysis of results of functional vascular analysis with outcome data of anti-angiogenic treatment. However, it is important to note that the study design allowed for separating the effect of anti-angiogenic treatment from that of chemotherapy.

## Conclusions

We demonstrate that the anti-VEGF antibody bevacizumab acutely impairs endothelial function of retinal arteries irrespective of blood pressure changes. Hence, functional imaging of the retinal microcirculation seems a valuable tool to monitor early effects of anti-VEGF treatment. Considering the close relationship between therapeutic and side effects of VEGF blockade, flicker provocation of retinal vessels may be a valid predictor of therapy success and outcome.

## Abbreviations

ABPM: 24-hour ambulatory blood pressure monitoring; DCE-MRI: Diffusion contrast enhanced MRI; HF: High-frequency; LF: Low-frequency; MTRS: Multiple trigonometric regressive spectral analysis; NO: Nitric oxide; VEGF: Vascular endothelial growth factor.

## Competing interests

The study was supported by the University Hospital Carl Gustav Carus. The funders had no role in study design, data collection and analysis, decision to publish, or preparation of the manuscript. Gunnar Folprecht received honoraries for lectures and advisory boards from Roche / Genentech. All other authors have declared that no competing interests exist. Additionally, Focke Ziemssen has received speaker’s fees or consultancy honoraria from Alcon, Alimera, Allergan, Biogen, Bayer, Pfizer and Novartis.

## Authors’ contribution

MR performed the statistical analysis and drafted the manuscript. RH assisted in the statistical analyses. TZ, FZ and GF designed the trial. TZ, KT, GF participated in the coordination of the trial and revised the manuscript for intellectual content. GE and HR revised the manuscript for intellectual content. All authors revised and approved the final manuscript.

## References

[B1] Miller K Wang M Gralow J Dickler M Cobleigh M Perez EA Shenkier T Cella D Davidson NE Paclitaxel plus bevacizumab versus paclitaxel alone for metastatic breast cancer N Engl J Med 2007 357 2666 2676 10.1056/NEJMoa072113 18160686

[B2] Sandler A Gray R Perry MC Brahmer J Schiller JH Dowlati A Lilenbaum R Johnson DH Paclitaxel-carboplatin alone or with bevacizumab for non-small-cell lung cancer N Engl J Med 2006 355 2542 2550 10.1056/NEJMoa061884 17167137

[B3] Longo R Sarmiento R Fanelli M Capaccetti B Gattuso D Gasparini G Anti-angiogenic therapy: rationale, challenges and clinical studies Angiogenesis 2002 5 237 256 10.1023/A:1024532022166 12906317

[B4] Escudier B Pluzanska A Koralewski P Ravaud A Bracarda S Szczylik C Chevreau C Filipek M Melichar B Bajetta E Bevacizumab plus interferon alfa-2a for treatment of metastatic renal cell carcinoma: a randomised, double-blind phase III trial Lancet 2007 370 2103 2111 10.1016/S0140-6736(07)61904-7 18156031

[B5] Hurwitz H Fehrenbacher L Novotny W Cartwright T Hainsworth J Heim W Berlin J Baron A Griffing S Holmgren E Bevacizumab plus irinotecan, fluorouracil, and leucovorin for metastatic colorectal cancer N Engl J Med 2004 350 2335 2342 10.1056/NEJMoa032691 15175435

[B6] Hurwitz H Saini S Bevacizumab in the treatment of metastatic colorectal cancer: safety profile and management of adverse events Semin Oncol 2006 33 S26 S34 17145522 10.1053/j.seminoncol.2006.08.001

[B7] Nalluri SR Chu D Keresztes R Zhu X Wu S Risk of venous thromboembolism with the angiogenesis inhibitor bevacizumab in cancer patients: a meta-analysis JAMA 2008 300 2277 2285 10.1001/jama.2008.656 19017914

[B8] Scappaticci FA Skillings JR Holden SN Gerber HP Miller K Kabbinavar F Bergsland E Ngai J Holmgren E Wang J Hurwitz H Arterial thromboembolic events in patients with metastatic carcinoma treated with chemotherapy and bevacizumab J Natl Cancer Inst 2007 99 1232 1239 10.1093/jnci/djm086 17686822

[B9] Facemire CS Nixon AB Griffiths R Hurwitz H Coffman TM Vascular endothelial growth factor receptor 2 controls blood pressure by regulating nitric oxide synthase expression Hypertension 2009 54 652 658 10.1161/HYPERTENSIONAHA.109.129973 19652084 PMC2746822

[B10] Mourad JJ des Guetz G Debbabi H Levy BI Blood pressure rise following angiogenesis inhibition by bevacizumab. A crucial role for microcirculation Ann Oncol 2008 19 927 934 10.1093/annonc/mdm550 18056916

[B11] Steeghs N Gelderblom H Roodt JO Christensen O Rajagopalan P Hovens M Putter H Rabelink TJ De Koning E Hypertension and rarefaction during treatment with telatinib, a small molecule angiogenesis inhibitor Clin Cancer Res 2008 14 3470 3476 10.1158/1078-0432.CCR-07-5050 18519779

[B12] Scartozzi M Galizia E Chiorrini S Giampieri R Berardi R Pierantoni C Cascinu S Arterial hypertension correlates with clinical outcome in colorectal cancer patients treated with first-line bevacizumab Ann Oncol 2009 20 227 230 18842611 10.1093/annonc/mdn637

[B13] Ziemssen T Reichmann H Cardiovascular autonomic testing in extrapyramidal disorders J Neurol Sci 2011 310 129 132 10.1016/j.jns.2011.07.032 21839477

[B14] Freeman R Wieling W Axelrod FB Benditt DG Benarroch E Biaggioni I Cheshire WP Chelimsky T Cortelli P Gibbons CH Consensus statement on the definition of orthostatic hypotension, neutrally mediated syncope and the postural tachycardia syndrome Clin Auton Res 2011 Apr 21 2 69 72 10.1007/s10286-011-0119-5 21431947

[B15] Vilser W Nagel E Lanzl I Retinal Vessel Analysis–new possibilities Biomed Tech (Berl) 2002 47 Suppl 1 Pt 2 682 685 12465273 10.1515/bmte.2002.47.s1b.682

[B16] Reimann M Prieur S Lippold B Bornstein SR Reichmann H Julius U Ziemssen T Retinal vessel analysis in hypercholesterolemic patients before and after LDL apheresis Atheroscler Suppl 2009 10 39 43 20129372 10.1016/S1567-5688(09)71808-2

[B17] Reimann M Julius U Bornstein SR Fischer S Reichmann H Rudiger H Ziemssen T Regular lipoprotein apheresis maintains residual cardiovascular and microvascular function in patients with advanced atherosclerotic disease Atherosclerosis Suppl 2013 14 135 141 10.1016/j.atherosclerosissup.2012.10.009 23357155

[B18] Ohkubo T Hozawa A Yamaguchi J Kikuya M Ohmori K Michimata M Matsubara M Hashimoto J Hoshi H Araki T Prognostic significance of the nocturnal decline in blood pressure in individuals with and without high 24-h blood pressure: the Ohasama study J Hypertens 2002 20 2183 2189 10.1097/00004872-200211000-00017 12409956

[B19] Schmidt C Berg D Prieur S Junghanns S Schweitzer K Globas C Schols L Reichmann H Ziemssen T Loss of nocturnal blood pressure fall in various extrapyramidal syndromes Movement disorder 2009 24 2136 2142 10.1002/mds.22767 19768815

[B20] Reimann M Friedrich C Gasch J Reichmann H Rudiger H Ziemssen T Trigonometric Regressive Spectral Analysis reliably Maps Dynamic Changes in Baroreflex Sensitivity and Autonomic Tone: The Effect of Gender and Age PLoS One 2010 5 e12187 doi:12110.11371/journal.pone.0012187 10.1371/journal.pone.0012187 20808439 PMC2922332

[B21] Ziemssen T Reimann M Gasch J Ruediger H Trigonometric Regressive Spectral Analysis An innovative tool for Evaluating the Autonomic Nervous System J Neural Transm 2013 in press 10.1007/s00702-013-1054-5 23812502

[B22] Gasch J Reimann M Reichmann H Rudiger H Ziemssen T Determination of baroreflex sensitivity during the modified Oxford maneuver by trigonometric regressive spectral analysis PLoS One 2011 6 e18061 10.1371/journal.pone.0018061 21437258 PMC3060917

[B23] Friedrich C Rüdiger H Schmidt C Herting B Prieur S Junghanns S Schweitzer K Globas C Schöls L Berg D Reichmann H Ziemssen T Baroreflex sensitivity and power spectral analysis during autonomic testing in different extrapyramidal syndromes Mov Disord 2010 25 3 315 24 10.1002/mds.22844 20014116

[B24] Lockhart AC Rothenberg ML Dupont J Cooper W Chevalier P Sternas L Buzenet G Koehler E Sosman JA Schwartz LH Phase I study of intravenous vascular endothelial growth factor trap, aflibercept, in patients with advanced solid tumors J Clin Oncol 2010 28 207 214 10.1200/JCO.2009.22.9237 19949018 PMC2815710

[B25] Morgan B Thomas AL Drevs J Hennig J Buchert M Jivan A Horsfield MA Mross K Ball HA Lee L Dynamic contrast-enhanced magnetic resonance imaging as a biomarker for the pharmacological response of PTK787/ZK 222584, an inhibitor of the vascular endothelial growth factor receptor tyrosine kinases, in patients with advanced colorectal cancer and liver metastases: results from two phase I studies J Clin Oncol 2003 21 3955 3964 10.1200/JCO.2003.08.092 14517187

[B26] Hamstra DA Rehemtulla A Ross BD Diffusion magnetic resonance imaging: a biomarker for treatment response in oncology J Clin Oncol 2007 25 4104 4109 10.1200/JCO.2007.11.9610 17827460

[B27] Murukesh N Dive C Jayson GC Biomarkers of angiogenesis and their role in the development of VEGF inhibitors Br J Cancer 2010 102 8 18 10.1038/sj.bjc.6605483 20010945 PMC2813747

[B28] Dorner GT Garhofer G Kiss B Polska E Polak K Riva CE Schmetterer L Nitric oxide regulates retinal vascular tone in humans Am J Physiol Heart Circ Physiol 2003 285 H631 H636 12750062 10.1152/ajpheart.00111.2003

[B29] Li B Ogasawara AK Yang R Wei W He GW Zioncheck TF Bunting S De Vos AM Jin H KDR (VEGF receptor 2) is the major mediator for the hypotensive effect of VEGF Hypertension 2002 39 1095 1100 10.1161/01.HYP.0000018588.56950.7A 12052848

[B30] Boegehold MA Heterogeneity of endothelial function within the circulation Curr Opin Nephrol Hypertens 1998 7 71 78 10.1097/00041552-199801000-00012 9442366

[B31] Kuo L Arko F Chilian WM Davis MJ Coronary venular responses to flow and pressure Circ Res 1993 72 607 615 10.1161/01.RES.72.3.607 8431988

[B32] Laham RJ Li J Tofukuji M Post M Simons M Sellke FW Spatial heterogeneity in VEGF-induced vasodilation: VEGF dilates microvessels but not epicardial and systemic arteries and veins Ann Vasc Surg 2003 17 245 252 10.1007/s10016-001-0299-x 12704544

[B33] Sica DA Angiogenesis inhibitors and hypertension: an emerging issue J Clin Oncol 2006 24 1329 1331 10.1200/JCO.2005.04.5740 16446321

[B34] Grothey A Sugrue MM Purdie DM Dong W Sargent D Hedrick E Kozloff M Bevacizumab beyond first progression is associated with prolonged overall survival in metastatic colorectal cancer: results from a large observational cohort study (BRiTE) J Clin Oncol 2008 26 5326 5334 10.1200/JCO.2008.16.3212 18854571

[B35] Vaklavas C Lenihan D Kurzrock R Tsimberidou AM Anti-vascular endothelial growth factor therapies and cardiovascular toxicity: what are the important clinical markers to target? Oncologist 2010 15 130 141 10.1634/theoncologist.2009-0252 20139170 PMC3227935

[B36] Yang JC Haworth L Sherry RM Hwu P Schwartzentruber DJ Topalian SL Steinberg SM Chen HX Rosenberg SA A randomized trial of bevacizumab, an anti-vascular endothelial growth factor antibody, for metastatic renal cancer N Engl J Med 2003 349 427 434 10.1056/NEJMoa021491 12890841 PMC2275324

[B37] Horowitz JR Rivard A van der Zee R Hariawala M Sheriff DD Esakof DD Chaudhry GM Symes JF Isner JM Vascular endothelial growth factor/vascular permeability factor produces nitric oxide-dependent hypotension. Evidence for a maintenance role in quiescent adult endothelium Arterioscler Thromb Vasc Biol 1997 17 2793 2800 10.1161/01.ATV.17.11.2793 9409257

